# Clinical outcomes of robot-assisted vs. conventional free-hand technique in spine surgery

**DOI:** 10.3389/fsurg.2025.1517470

**Published:** 2025-08-22

**Authors:** Hui Yan, Yuxuan Wu, Xiaokang Cheng, Chunyang Xu, Tianci Yang, Beixi Bao

**Affiliations:** Department of Orthopedics, Beijing Tongren Hospital, Capital Medical University, Beijing, China

**Keywords:** robot-assisted surgery, conventional free-hand surgery, postoperative recovery, generalized estimating equation (GEE), spinal surgery

## Abstract

**Background:**

Robot-assisted surgery has been increasingly applied in spinal surgery in recent years, but the differences in efficacy compared to conventional free-hand surgery remain unclear. This study aims to evaluate the impact of these two surgical approaches on spinal surgery patients by analyzing baseline characteristics, surgical data, short-term postoperative outcomes, and long-term functional recovery and pain relief.

**Methods:**

This study first analyzed the differences in baseline characteristics and surgical data between the robot-assisted and conventional free-hand surgery groups, including age, gender, diabetes, hypertension, smoking, and alcohol consumption. Multivariate logistic regression was then used to explore the effects of baseline characteristics and surgical methods on short-term postoperative outcomes, such as complications, reoperations, fracture healing, and spinal alignment recovery. Finally, generalized estimating equations (GEE) were employed to assess the impact of surgical methods on long-term postoperative outcomes, including Oswestry Disability Index (ODI) and pain scores.

**Results:**

Diabetes and hypertension significantly increased the risk of postoperative complications and reoperation, while robot-assisted surgery significantly reduced the incidence of complications and reoperation. In terms of spinal structural recovery after surgery, the robot-assisted surgery group showed better results. Long-term follow-up revealed that robot-assisted surgery significantly reduced ODI and pain scores, and over time, the robot-assisted group consistently demonstrated superior functional recovery and pain relief compared to the conventional surgery group.

**Conclusion:**

Robot-assisted surgery showed significant advantages in both short-term postoperative recovery and long-term functional improvement and pain relief. It outperformed conventional free-hand surgery in reducing complication rates, accelerating postoperative recovery, lowering reoperation rates, and promoting fracture healing and spinal alignment recovery.

## 1 Introduction

Spinal diseases are one of the leading causes of disability and pain worldwide, affecting the quality of life for millions of patients (1, 2). With advancements in medical technology, spinal surgery has become an essential treatment for severe spinal conditions, aiming to correct anatomical structures, relieve nerve compression, and stabilize the spine (3, 4). These interventions help alleviate pain, improve function, and restore the patient's ability to perform daily activities. Conventional free-hand surgery is widely used in clinical practice, and there is a wealth of experience in its application (5). However, the complexity of the procedure and its reliance on the surgeon's precision often limit the consistency of surgical outcomes. Additionally, conventional surgery is often associated with significant blood loss, prolonged surgical time, and a higher risk of complications, posing challenges to postoperative recovery (6).

In recent years, the introduction of robot-assisted surgery has provided a more precise and stable approach to spinal surgery (7, 8). Robot-assisted surgery, by offering high-precision three-dimensional imaging and enhanced operational flexibility, enables surgeons to better avoid critical structures and accurately target the surgical site, thus reducing surgical errors, shortening operating times, and accelerating recovery (9). Moreover, robotic technology may also reduce intraoperative blood loss, lower the incidence of postoperative complications, and improve postoperative functional recovery (10). While robotic surgery offers numerous advantages, it also faces several challenges. Firstly, the high cost of robotic surgical equipment and the need for specialized infrastructure make it difficult for many hospitals to implement large-scale adoption. Secondly, the complexity of the surgery itself requires that surgeons not only have extensive clinical experience but also master the corresponding operational skills. Additionally, the maintenance and updating of robotic systems present a continuous financial burden.

The Oswestry Disability Index (ODI) and pain scores are key indicators for evaluating the efficacy of spinal surgery (11). The ODI quantifies the level of functional impairment in daily life and is a commonly used scale for assessing functional recovery after spinal surgery (12). Pain scores are used to evaluate the relief of postoperative pain, which is an important metric for measuring the patient's quality of life after surgery. Other important clinical outcomes in spinal surgery include fracture healing status, spinal alignment recovery, spinal cord compression relief, and nerve root compression relief, all of which provide a comprehensive picture of the patient's postoperative recovery progress.

However, existing studies comparing the efficacy of robot-assisted surgery and conventional free-hand surgery are relatively limited, and the results have been inconsistent. Some studies have shown that robot-assisted surgery can significantly shorten operating time, reduce blood loss, and lower complication rates, while others suggest that the long-term benefits of robot-assisted surgery in terms of functional recovery and pain relief remain unclear (13, 14). Therefore, this study aims to systematically evaluate the clinical outcomes of both surgical methods by comparing preoperative baseline characteristics, surgical data, short-term follow-up results, and long-term functional recovery. Through this research, we hope to provide clearer evidence-based guidance for the selection of spinal surgery techniques and further promote the application and development of robotic technology in spinal surgery.

## 2 Materials and methods

### 2.1 Study subjects

This retrospective cohort study included patients who underwent spinal surgery between January 2021 and January 2024 at our hospital. The subjects included patients who received either robot-assisted surgery (Robot-Assisted Surgery) or conventional free-hand surgery (Conventional Free-Hand Surgery) for spinal procedures. Inclusion criteria: (1) Age ≥ 18 years; (2) Patients who underwent either robot-assisted surgery or conventional freehand surgery; (3) Complete follow-up data. Exclusion criteria: (1) Presence of severe systemic diseases or inability to tolerate surgery; (2) History of previous spinal surgery; (3) Severe psychiatric disorders or conditions affecting follow-up compliance; (4) Missing key data. All patients underwent a detailed preoperative clinical evaluation, including medical history, physical examination, and imaging studies.

### 2.2 Data collection

The data for this study were obtained from patients' hospital records and postoperative follow-up records. The collected data included baseline characteristics (age, gender, BMI, diabetes, hypertension, smoking, and drinking status), surgical data (type of surgery, surgery duration, intraoperative blood loss, and occurrence of complications), and postoperative follow-up data. The follow-up time points were 1 month, 3 months, and 6 months after surgery. Long-term follow-up indicators included the Oswestry Disability Index (ODI), pain level, reoperation rates, and time to return to work. Short-term follow-up indicators included the occurrence of complications, length of hospital stay, fracture healing status, spinal alignment recovery, spinal cord compression relief, and nerve root compression relief. Pain levels were assessed using the Visual Analog Scale (VAS). Fracture healing status, spinal alignment recovery, spinal cord compression status, and nerve root compression status were evaluated through imaging studies. In addition, the main costs associated with robotic-assisted surgery were collected, which include three major components: equipment costs, annual maintenance and consumables costs, and training costs. The cost of the Da Vinci system equipment is $2.5 million (including installation and initial calibration fees). The annual maintenance and consumables costs total $100,000 per year, and the training costs are $10,000 per year. Therefore, the initial total investment is $2.5 million, and the annual operating cost is $110,000.

### 2.3 Statistical analysis

All data were statistically analyzed using R 4.4.1 software. Continuous variables were expressed as medians (minimum-maximum) and compared between groups using the independent sample *t*-test or Mann–Whitney *U* test. Categorical variables were expressed as frequencies and percentages, and comparisons between groups were performed using the Chi-square test or Fisher's exact test. To evaluate the impact of the surgical method on postoperative outcomes, descriptive analysis was first conducted on baseline characteristics and surgical data. Multivariate logistic regression was then used to assess the effects of baseline characteristics and surgical methods on the occurrence of postoperative complications, reoperation, fracture healing, and spinal alignment recovery.

To further assess the impact of robot-assisted surgery vs. conventional free-hand surgery on long-term functional recovery and pain relief, the Generalized Estimating Equations (GEE) model was used to analyze changes in ODI and pain scores over time (15). In this model, surgical method, follow-up time, and their interactions were included as independent variables, while ODI and pain scores were used as dependent variables. A *P*-value < 0.05 was considered statistically significant.

## 3 Results

### 3.1 Baseline characteristics and surgical data of the two surgical methods

We first analyzed the differences in baseline characteristics and surgical data between the robot-assisted surgery and conventional free-hand surgery groups. The results showed no significant differences between the two groups in terms of age, gender, diabetes, hypertension, smoking, and drinking status. The preoperative ODI score was higher in the robot-assisted surgery group (45.05 vs. 42.53, *P* = 0.0348). The proportions of decompression surgery and fusion surgery were higher in the robot-assisted surgery group, while the proportion of corrective surgery was lower. The operation duration was slightly shorter (3.07 h vs. 3.52 h, *P* = 0.0393), and the intraoperative blood loss was less (436.31 ml vs. 475.59 ml, *P* = 0.0268) (Table 1).

**Table 1:** Baseline information and surgery-related data of patients undergoing robot-assisted surgery and conventional free-hand surgery.

Variables	All Patients (*n* = 180)	Robot-Assisted (*n* = 80)	Conventional (*n* = 100)	*P*-values
Age	48 (18-75)	47 (21–75)	48 (18–72)	0.508
Gender				0.129815
Male	106 (58.89%)	42 (52.5%)	64 (64%)	
Female	74 (41.11%)	38 (47.5%)	36 (36%)	
Diabetes				0.2471286
Yes	21 (11.67%)	12 (15%)	9 (9%)	
No	159 (88.33%)	68 (85%)	91 (91%)	
Hypertension				0.369044
Yes	40 (22.22%)	15 (18.75%)	25 (25%)	
No	140 (77.78%)	65 (81.25%)	75 (75%)	
Smoking				0.32297
Yes	31 (17.22%)	11 (13.75%)	20 (20%)	
No	149 (82.78%)	69 (86.25%)	80 (80%)	
Drinking				0.739198
Yes	50 (27.78%)	21 (26.25%)	29 (29%)	
No	130 (72.22%)	59 (73.75%)	71 (71%)	
Preoperative Oswestry Disability Index (ODI)	44.15 (26.84–62.48)	45.05 (27.09–62.48)	42.53 (26.84–61.95)	0.0348
Preoperative Pain Level	6 (5–8)	6 (5–7)	6 (5–8)	0.784
Surgical Type				0.01744933
Decompressive Surgery	85 (47.22%)	41 (51.25%)	44 (44%)	
Fusion Surgery	75 (41.67%)	36 (45%)	39 (39%)	
Corrective Surgery	20 (11.11%)	3 (3.75%)	17 (17%)	
Surgical Duration (hour)	3.31 (1.53–4.98)	3.07 (1.53–4.98)	3.52 (1.56–4.97)	0.0393
Intraoperative Blood Loss (ml)	464.21 (66.50–806.00)	436.31 (66.50–753.78)	475.59 (71.72–806.00)	0.0268

### 3.2 Differences in short-term postoperative follow-up performance between the two surgical methods

The results showed that the incidence of complications (2.5% vs. 11%, *P* = 0.0402), length of hospital stay (median 4 days vs. 5 days, *P* = 0.0421), time to return to work (median 63 days vs. 77 days, *P* = 0.0018), and reoperation rates (2.5% vs. 13%, *P* = 0.0133) were significantly lower in the robot-assisted surgery group than in the conventional free-hand surgery group. Additionally, the robot-assisted surgery group had lower postoperative ODI scores (27.84 vs. 30.27, *P* = 0.0044), lower postoperative pain scores (4 vs. 5, *P* = 0.0194), higher fracture healing rates (93.75% vs. 79%, *P* = 0.0054), and higher spinal alignment recovery rates (96.25% vs. 87%, *P* = 0.0356). The relief rates of spinal cord compression and nerve root compression were also higher in the robot-assisted surgery group, at 98.75% and 97.5%, respectively, compared to 87% and 85% in the conventional surgery group (*P* < 0.01). These results indicate that robot-assisted surgery offers superior short-term recovery compared to conventional free-hand surgery (Table 2).

**Table 2:** Postoperative short-term follow-up information.

Variables	All Patients (*n* = 180)	Robot-Assisted (*n* = 80)	Conventional (*n* = 100)	*P*-values
Complications Occurrence				0.0402
Yes	13 (7.22%)	2 (2.5%)	11 (11%)	
No	167 (92.78%)	78 (97.5%)	89 (89%)	
Length of Hospital Stay (day)	4 (2–7)	4 (2–6.5)	5 (3.5–7)	0.0421
Time to Return to Work (day)	68 (14–118)	63 (16–118)	77 (14–115)	0.0018
Reoperation				0.0133
Yes	15 (8.33%)	2 (2.5%)	13 (13%)	
No	165 (91.67%)	78 (97.5%)	87 (87%)	
Postoperative Oswestry Disability Index	28.92 (18.88–38.13)	27.84 (18.88–37.69)	30.27 (19.11–38.13)	0.0044
Postoperative Pain Level	4 (3–6)	4 (3–5)	5 (3–6)	0.0194
Fracture Healing Status				0.0054
Healed	154 (85.56%)	75 (93.75%)	79 (79%)	
Not Healed	26 (14.44%)	5 (6.25%)	21 (21%)	
Spinal Alignment Recovery				0.0356
Normal Alignment	164 (91.11%)	77 (96.25%)	87 (87%)	
Abnormal Alignment	16 (8.89%)	3 (3.75%)	13 (13%)	
Spinal Cord Compression Status				0.0037
No Compression	166 (92.22%)	79 (98.75%)	87 (87%)	
Compression	14 (7.78%)	1 (1.25%)	13 (13%)	
Nerve Root Compression Status				0.0042
No Compression	163 (90.56%)	78 (97.5%)	85 (85%)	
Compression	17 (9.44%)	2 (2.5%)	15 (15%)	

### 3.3 The impact of baseline information and surgical methods on short-term postoperative recovery

In terms of complications, diabetes (OR = 5.127, *P* = 0.001) and hypertension (OR = 3.818, *P* = 0.006) significantly increased the risk of complications, while robot-assisted surgery significantly reduced the occurrence of complications (OR = 0.057, *P* = 0.041). Reoperation rates were significantly influenced by diabetes (OR = 1.842, *P* = 0.001) and hypertension (OR = 6.478, *P* = 0.023), with robot-assisted surgery significantly reducing the likelihood of reoperation (OR = 0.019, *P* = 0.019). In terms of fracture healing status, diabetes (OR = 0.03, *P* = 0.001), hypertension (OR = 0.021, *P* = 0.007), and smoking (OR = 0.062, *P* = 0.026) significantly impacted fracture healing, while robot-assisted surgery showed some advantage over conventional free-hand surgery in this regard (OR = 16.309, *P* = 0.096). For spinal alignment recovery, hypertension (OR = 0.05, *P* = 0.025) and smoking (OR = 0.02, *P* = 0.015) significantly inhibited spinal alignment recovery, while robot-assisted surgery had a strong positive impact, though it did not reach strict statistical significance (*P* = 0.057). Regarding spinal cord compression status, diabetes (OR = 0.15, *P* = 0.026) and smoking (OR = 0.34, *P* = 0.045) negatively affected recovery from spinal cord compression, and robot-assisted surgery did not show a significant effect on recovery. High preoperative ODI scores also reduced the likelihood of recovery from spinal cord compression (OR = 0.656, *P* = 0.058), though it did not reach statistical significance. Lastly, for nerve root compression status, diabetes (OR = 0.012, *P* = 0.004), smoking (OR = 0.031, *P* = 0.006), and preoperative ODI scores (OR = 0.836, *P* = 0.018) were unfavorable factors for recovery, while robot-assisted surgery showed a better effect in relieving nerve root compression compared to conventional free-hand surgery (OR = 4.577, *P* = 0.029) (Table 3).

**Table 3:** Multivariate logistic regression analysis of the impact of surgical methods and baseline information on treatment outcomes.

Postoperative outcomes	Outputs	Age	Gender	Diabetes	Hypertension	Smoking	Drinking	PRE ODI	PRE Pain	Surgical Techniques
Complications Occurrence	*p* value	0.573	0.508	0.001	0.006	0.493	0.885	0.548	0.925	0.041
	OR	0.982	2.066	5.127	3.818	2.450	1.195	0.959	0.935	0.057
	CI_lower	0.924	0.241	1.490	2.886	0.190	0.108	0.835	0.232	0.004
	CI_upper	1.045	17.699	10.638	5.052	31.653	13.230	1.100	3.766	0.884
Reoperation	*p* value	0.486	0.285	0.000	0.023	0.328	0.505	0.198	0.100	0.019
	OR	0.978	0.300	1.842	6.478	3.603	0.455	1.070	2.929	0.019
	CI_lower	0.919	0.033	1.628	1.420	0.276	0.045	0.965	0.813	0.001
	CI_upper	1.041	2.729	2.027	12.788	47.010	4.598	1.186	10.555	0.521
Fracture Healing Status	*p* value	0.604	0.659	0.001	0.007	0.026	0.365	0.334	0.220	0.096
	OR	0.984	1.706	0.030	0.021	0.062	0.314	0.929	0.289	16.309
	CI_lower	0.926	0.159	0.001	0.001	0.005	0.026	0.801	0.040	10.641
	CI_upper	1.046	18.273	0.061	0.341	0.722	3.855	1.078	2.107	23.642
Spinal Alignment Recovery	*p* value	0.086	0.585	0.050	0.025	0.015	0.239	0.113	0.099	0.057
	OR	0.910	2.416	0.010	0.048	0.019	0.149	0.860	0.042	16.265
	CI_lower	0.817	0.102	0.000	0.000	0.000	0.006	0.714	0.001	10.867
	CI_upper	1.013	57.356	0.988	0.503	0.297	3.539	1.036	1.820	21.497
Spinal Cord Compression Status	*p* value	0.961	0.730	0.026	0.621	0.045	0.255	0.058	0.179	0.080
	OR	0.997	1.977	0.150	0.379	0.340	0.094	0.656	0.127	22.123
	CI_lower	0.888	0.041	0.000	0.008	0.000	0.002	0.424	0.006	10.291
	CI_upper	1.120	95.468	0.401	17.722	0.821	5.518	1.015	2.564	35.480
Nerve Root Compression Status	*p* value	0.448	0.620	0.004	0.065	0.006	0.072	0.018	0.442	0.029
	OR	1.033	2.086	0.012	0.083	0.031	0.101	0.836	0.443	4.577
	CI_lower	0.950	0.114	0.001	0.006	0.003	0.008	0.721	0.055	1.446
	CI_upper	1.123	38.074	0.236	1.163	0.367	1.228	0.970	3.535	8.849

### 3.4 Long-term changes in postoperative ODI and pain scores

**Figure 1:**
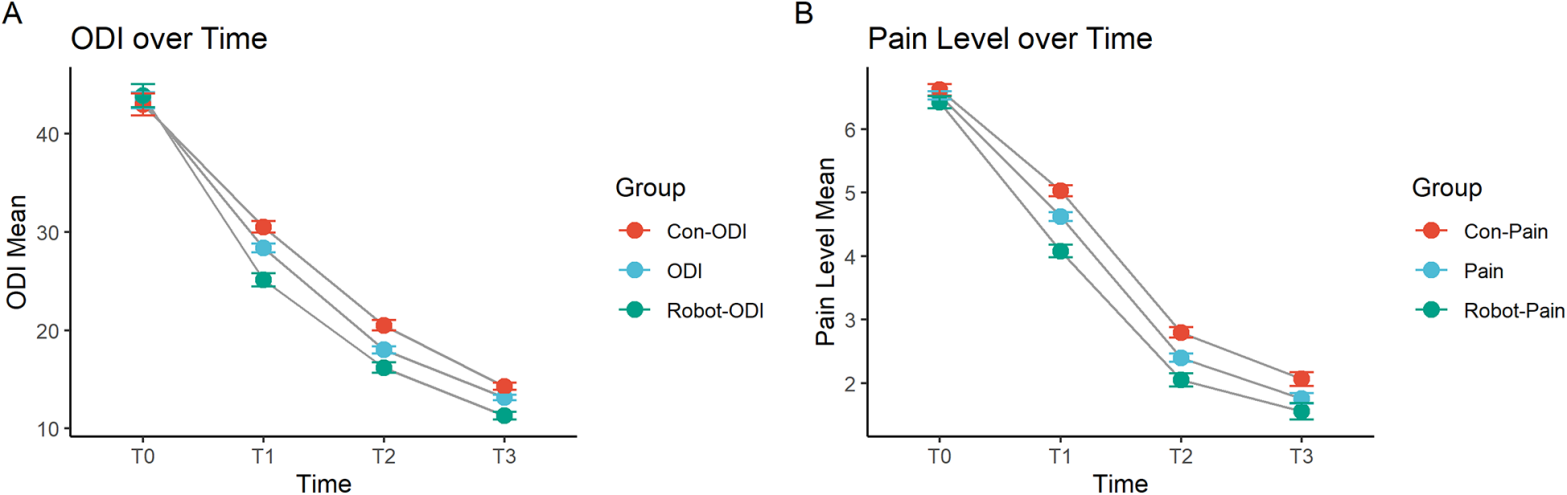
Changes in **(A)** ODI and **(B)** pain scores at 1 month, 3 months, and 6 months post-surgery. T0: Preoperative, T1: 1 month post-surgery, T2: 3 months post-surgery, T3: 6 months post-surgery.

We used line graphs to analyze the long-term changes in ODI and pain scores after surgery. The results showed that both the robot-assisted surgery group and the conventional free-hand surgery group experienced significant decreases in ODI and pain scores at all time points after surgery. At 1 month (T1) and 3 months (T2) postoperatively, the robot-assisted surgery group had significantly lower ODI and pain scores than the conventional surgery group. By 6 months (T3) postoperatively, the ODI and pain scores in both groups became closer. Therefore, the robot-assisted surgery group demonstrated better functional recovery and pain relief than the conventional free-hand surgery group, with more pronounced effects at 1 month and 3 months postoperatively (Figures 1A,B).

### 3.5 Evaluation of the long-term impact of robot-assisted surgery on patient outcomes using generalized estimating equations

Next, we evaluated the long-term impact of robot-assisted surgery on patient outcomes. For the ODI scores, the B value for the Group (Robot-Assisted) was −39.75, with a *P* value of 0, indicating that compared to conventional free-hand surgery, robot-assisted surgery significantly reduced the ODI scores, indicating a reduction in postoperative disability. At T1 (one month after surgery), the B value was −4.76, with a *P* value of 0.00095, showing that over time, ODI scores significantly decreased, reflecting improvements in functional recovery. The B values at T2 (three months after surgery) and T3 (six months after surgery) were −6.02 and −6.72, respectively, with *P* values of 0.0187 and 0.00000133, demonstrating that as time progressed, ODI scores continued to decrease, with sustained functional improvements. The interaction between surgical technique and time showed that the B value for Surgical TechniquesTimeT1 was −27.1*,* with a *P* value of 0.0017, indicating a significant interaction between robot-assisted surgery and time, with the robot-assisted group showing a more pronounced decrease in ODI scores during the early postoperative period. The B values for Surgical TechniquesTimeT2 and TimeT3 were −45.3 and −42.5, respectively, with *P* values of 0, suggesting that during longer follow-up periods, the ODI scores continued to decrease significantly in the robot-assisted surgery group, with functional recovery being markedly better than in the conventional surgery group.

For postoperative pain scores, the B value for the Group (Robot-Assisted) was −6.69, with a *P* value of 8.20 × 10^−6^, indicating that robot-assisted surgery significantly reduced postoperative pain scores, providing better pain relief compared to conventional surgery. The B value at one month post-surgery was −2.22, with a *P* value of 0.2598, indicating that pain levels decreased at one month but did not reach statistical significance. At three months post-surgery, the B value was −4.98, with a *P* value of 0.0223, showing a significant reduction in pain scores, with noticeable pain relief for patients as time progressed. At six months post-surgery, the B value was −4.07, with a *P* value of 0.0504, which was close to statistical significance, indicating that pain scores continued to decrease at later time points, though the reduction was slightly less significant statistically. The interaction between surgical techniques and time showed that the B value for Surgical Techniques*TimeT1 was −8.69, with a *P* value of 0.0215, indicating that at early time points, robot-assisted surgery provided significantly better pain relief than conventional surgery. The B values for three and six months post-surgery were −12.73 and −13.48, with *P* values of 0.0106 and 3.90 × 10^−11^, respectively, indicating that during longer follow-up periods, the robot-assisted surgery group continued to experience significantly better pain relief than the conventional surgery group (Table 4).

**Table 4:** Evaluation of the impact of surgical methods on long-term postoperative recovery using generalized estimating equations.

Term	ODI			Pain Level		
	B	*P*-value	SE	B	*P*-value	SE
Group						
Robot-Assisted	−39.75	0.00	4.26	−6.69	8.20 × 10^−6^	1.5
Time						
T1	−4.76	0.00	1.44	−2.22	0.2598	1.97
T2	−6.02	0.02	2.56	−4.98	0.0223	2.18
T3	−6.72	0.00	1.39	−4.07	0.0504	2.08
Group*Time						
Surgical Techniques*TimeT1	−27.1	0.00	8.63	−8.69	0.0215	3.78
Surgical Techniques*TimeT2	−45.3	0.00	2.65	−12.73	0.0106	4.98
Surgical Techniques*TimeT3	−42.5	0.00	4.34	−13.48	3.90E-11	2.04

In summary, robot-assisted surgery significantly improved postoperative functional recovery and pain relief, particularly at various time points following surgery. Over time, both ODI and pain scores showed significant reductions, with robot-assisted surgery demonstrating superior outcomes compared to conventional surgery.

### 3.6 Cost-benefit analysis of robotic-assisted surgery

**Figure 2:**
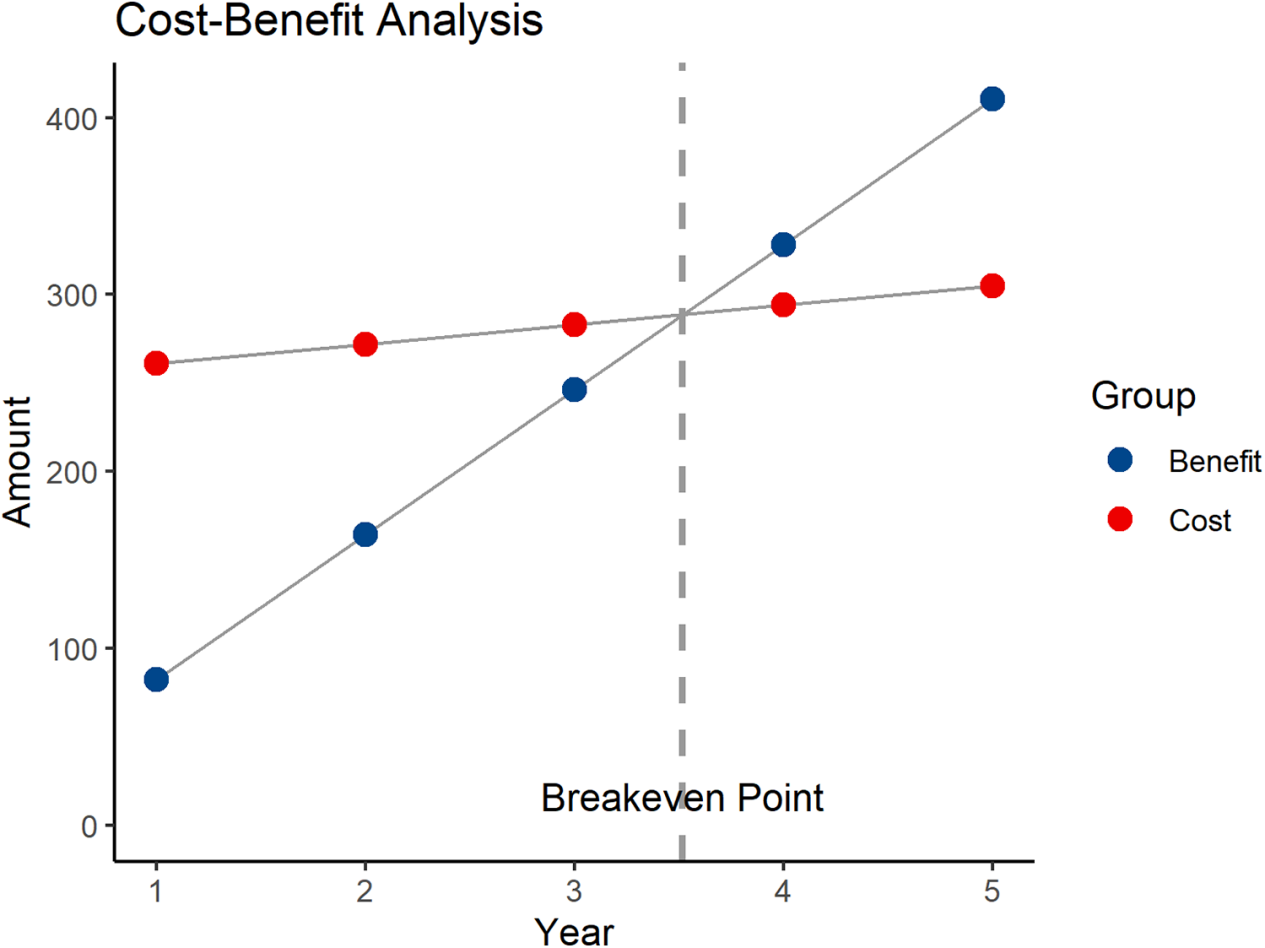
Cost-benefit analysis of robotic surgery.

**Figure 3:**
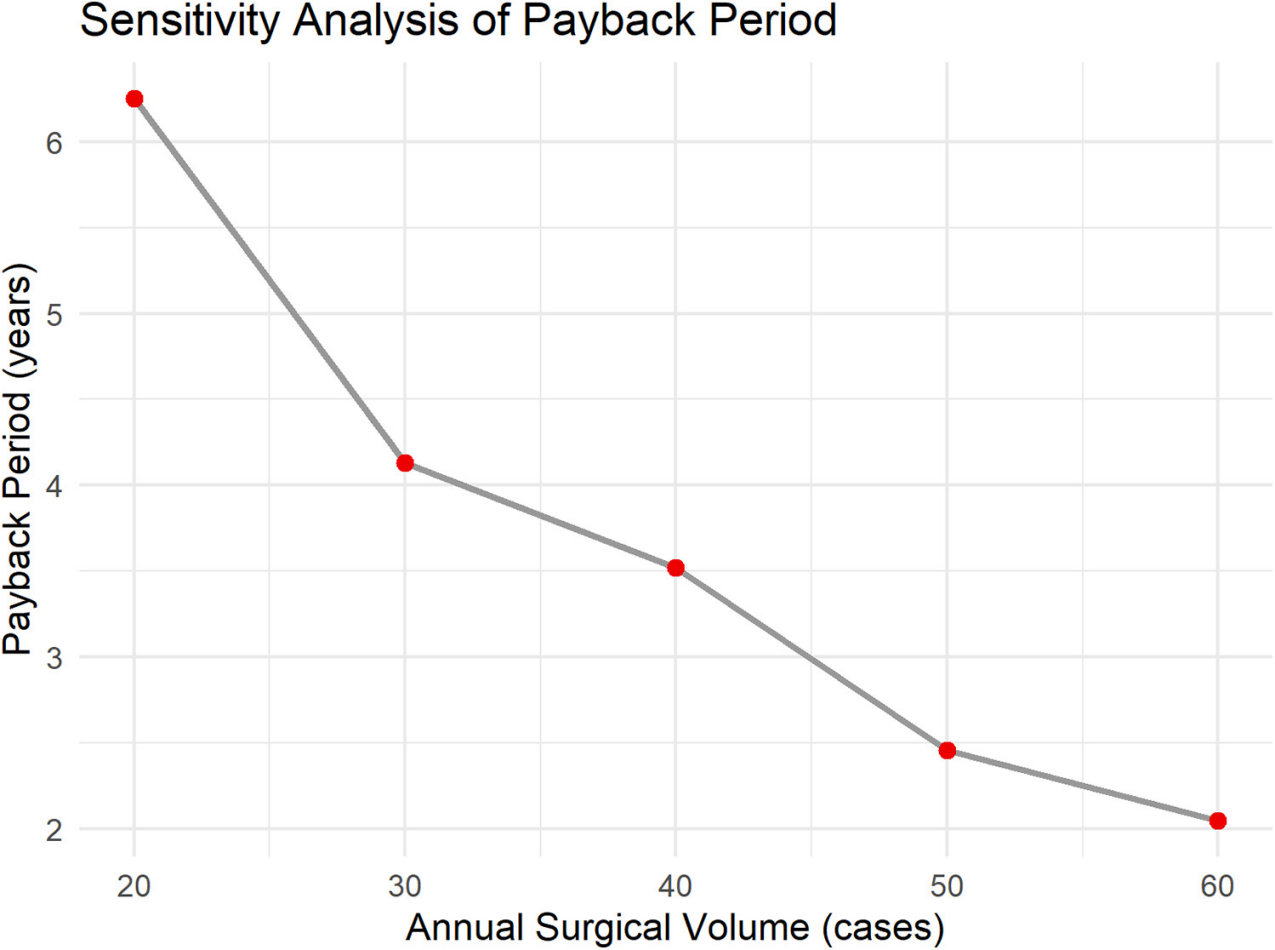
Sensitivity analysis of payback period.

The benefits primarily include three components: the cost reduction due to shortened patient hospital stays, the reduction in treatment costs for patient complications, and the increased surgical fees. As per previous analysis, robotic-assisted surgery can reduce the average hospital stay by 2 days. With approximately 40 patients undergoing robotic-assisted surgery each year, and an average daily hospital cost of $200, the reduced hospitalization cost is: 2 * 40 * 200 = $16,000. Each year, complications are reduced by 5 cases, with the treatment cost for each complication being $1,000. The reduced complication treatment cost is: 5 * 1,000 = $5,000. The surgical fee is $20,000 per procedure, and the total surgical fees for 40 procedures are: 20,000 * 40 = $800,000. The total annual benefit is $821,000. The annual net benefit is: total annual benefit—annual operating cost, i.e., $821,000–$110,000 = $711,000. The payback period is $2.5 million/$711,000 ≈ 3.52 years (Figure 2). To further verify the robustness of the economic evaluation, a sensitivity analysis was conducted. The results show that when the annual surgical volume is 20, 30, 40, 50, and 60 cases, the corresponding payback periods are approximately 6.25 years, 4.13 years, 3.52 years, 2.46 years, and 2.05 years, respectively (Figure 3). As the surgical volume increases, the payback period significantly shortens.

### 3.7 Stratified analysis

Given the significant baseline differences in preoperative ODI scores and surgical types between the two groups, we performed separate stratified analyses for these two variables. Patients were divided into high-ODI and low-ODI groups based on the median preoperative ODI score. The results showed that in both the high-ODI and low-ODI subgroups, the postoperative ODI scores were significantly lower in the robot-assisted surgery group compared to the conventional freehand surgery group. Similarly, among patients undergoing different types of surgery, the robot-assisted group consistently demonstrated significantly lower postoperative ODI scores than the conventional surgery group (Supplementary Figures 1A–E). This indicates that baseline differences did not significantly interfere with the intervention effect.

## 4 Discussion

This study provides a comprehensive evaluation of the clinical outcomes of robot-assisted surgery vs. conventional free-hand surgery in spinal surgery by comparing their efficacy in both short-term and long-term follow-ups. The results show that robot-assisted surgery has significant advantages in reducing intraoperative blood loss, shortening surgery time, reducing postoperative complications, and improving functional recovery.

Robotic-assisted surgery allows for precise preoperative planning and real-time intraoperative imaging guidance. The robotic arm executes drilling and screw placement strictly according to the planned trajectory, minimizing angular deviations caused by manual manipulation. This significantly improves the accuracy of pedicle screw placement, with errors controlled within sub-millimeter levels, thereby reducing screw misplacement (16, 17). As a result, injury to surrounding soft tissues and blood vessels is minimized, leading to reduced intraoperative blood loss. Moreover, reduced tissue damage contributes to a lower incidence of postoperative complications. The accurate screw trajectory also helps to avoid injury to the spinal cord, nerve roots, or adjacent vessels. Multiple studies have confirmed that robotic systems offer significant advantages in improving surgical precision and safety, ultimately leading to better perioperative outcomes.

Multivariate logistic regression analysis showed that diabetes and hypertension are important factors affecting postoperative recovery. They mainly delay bone healing and spinal restoration by impairing blood circulation and immune function, resulting in prolonged recovery time and increased complications (18, 19, 20). Moreover, our findings suggest that although some indicators did not reach strict statistical significance, robot-assisted surgery significantly reduced the rates of complications and reoperation and showed advantages in bone healing and spinal alignment recovery. This suggests that robotic surgery has potential clinical advantages in terms of precision and safety, improving postoperative recovery outcomes (21). While the lack of statistical significance may be due to limitations in sample size or follow-up duration, these trends remain clinically significant, highlighting the need for further research to confirm these preliminary findings and provide a basis for broader clinical applications in the future.

In long-term follow-ups, the ODI and pain scores of the robot-assisted surgery group significantly decreased at all time points after surgery, especially at one month and three months postoperatively, where the improvement was more pronounced in the robot group. This suggests that robot-assisted surgery has a clear early advantage in promoting postoperative functional recovery. At six months, although the ODI and pain scores of both groups converged, the robot group still showed better long-term recovery. Thus, robot-assisted surgery not only improved functional recovery and pain relief in the short term but also had a positive impact on patients' long-term prognosis. Using generalized estimating equations to further assess the relationship between surgical methods and long-term postoperative outcomes, the results showed that robot-assisted surgery significantly reduced ODI and pain scores, indicating that this surgical method effectively reduces postoperative disability and pain. Particularly in the early stages after surgery, the robot-assisted surgery group exhibited more significant reductions in ODI and pain scores, demonstrating robust functional recovery. The interaction analysis between surgical method and time also confirmed that robot-assisted surgery consistently showed better outcomes during follow-up, especially at one and three months postoperatively. Its minimally invasive and high-precision characteristics significantly reduced postoperative pain, accelerated recovery, and continued to show efficacy in long-term follow-ups. This technology not only improved patients' quality of life but also advanced spinal surgery, holding great clinical significance and application value (22).

Inexperienced surgeons may require more time to adapt to robot-assisted surgery initially, leading to longer operative times. Studies have shown that in total hip arthroplasty, robot-assisted surgery initially takes about 9–13 min longer than conventional surgery, and surgeons usually need to perform 12–17 cases to improve efficiency (23). Therefore, a systematic training program is necessary to help surgeons overcome challenges and improve technical proficiency.

Despite providing strong evidence for the efficacy of robot-assisted surgery compared to conventional free-hand surgery, this study has some limitations. First, as a retrospective study, the accuracy of the data may be affected by recording bias (24). Second, the relatively small sample size and the extended data collection period may limit the applicability of the results to a broader population and reduce the statistical power of the analysis. A six-month follow-up period is relatively short for spine surgery and may not fully reflect long-term surgical outcomes or delayed complications. Future studies should be prospective, larger in scale, with shorter data collection intervals and longer follow-up durations, in the form of randomized controlled trials, to further validate the findings of this study and enhance their external validity and statistical power.

## 5 Conclusion

This study shows that compared with traditional free surgery, the application of robot assisted surgery in spinal surgery significantly reduces postoperative complications, reoperation rates, and postoperative disability levels, while effectively reducing patients' postoperative pain. Robot assisted surgery has shown significant advantages in promoting early and long-term functional recovery after surgery. However, due to limitations in sample size and research scope, larger scale studies and long-term follow-up are still needed to further validate the conclusions of this study.

## Data Availability

The raw data supporting the conclusions of this article will be made available by the authors, without undue reservation.
