# Supplementary figures and images for: Clinical outcomes of robot-assisted vs. conventional free-hand technique in spine surgery
Source: Front Surg. 2025 Aug 22;12:1517470. doi: 10.3389/fsurg.2025.1517470 (PMC12411553; doi:10.3389/fsurg.2025.1517470)

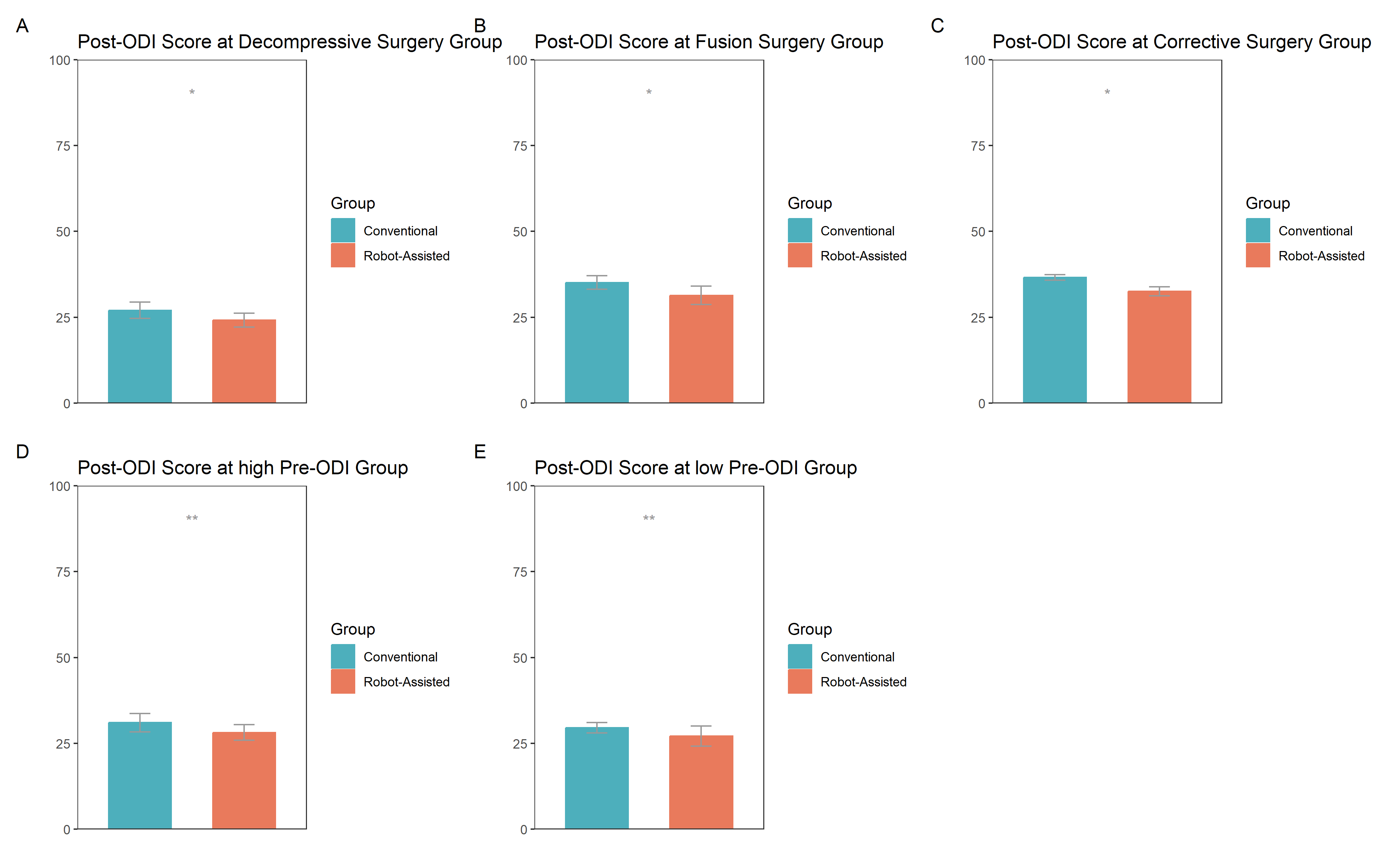

Supplement: Supplementary file 1 [file Image1.tif]
